# The PG-SGA outperforms the NRS 2002 for nutritional risk screening in cancer patients: a retrospective study from China

**DOI:** 10.3389/fnut.2023.1272420

**Published:** 2023-11-22

**Authors:** Xinqiao Chen, Xiangliang Liu, Wei Ji, Yixin Zhao, Yuwei He, Yining Liu, Qiguang Li, Hanping Shi, Jiuwei Cui

**Affiliations:** ^1^Cancer Center, The First Affiliated Hospital of Jilin University, Changchun, China; ^2^Beijing Shijitan Hospital, Capital Medical University, Beijing, China

**Keywords:** nutritional risk screening, PG-SGA, cancer patients, retrospective study, NRS 2002

## Abstract

**Background and aims:**

As a chronic wasting disease, cancer can lead to metabolic and physiological changes in patients, resulting in severe malnutrition. Therefore, accurate assessment of nutritional status and adoption of scientifically sound nutritional interventions are of great importance for patients with cancer. This study aimed to assess the necessity of implementing the Nutrition Risk Screening 2002 (NRS 2002) tool in conjunction with the Patient-Generated Subjective Global Assessment (PG-SGA) in patients with cancer.

**Methods:**

This retrospective study collected the clinical data of cancer patients from November 2011 to December 2018 in the Department of Oncology, Cancer Center, First Hospital of Jilin University. The NRS 2002 and the PG-SGA were used as screening tools for malnutrition. Clinical characteristics and laboratory results were detected. Anthropometric indices including hand-grip strength (HGS), visceral fat area (VFA), calf circumstance (CC), and appendicular skeletal muscle mass index (ASMI) were also collected. The diagnostic results from the NRS 2002 were compared to the malnutrition diagnosis using the PG-SGA.

**Results:**

Of the 2,645 patients included in this retrospective study, the nutritional risk was found in 1763 (66.6%) patients based on the PG-SGA, and in 240 (9.1%) patients based on the NRS 2002, respectively. Among the 240 patients evaluated by the NRS 2002 for risk of malnutrition, 230 were also assessed by the PG-SGA as malnourished. There were no significant differences observed in the clinical characteristics and laboratory parameters between the two groups.

**Conclusion:**

The PG-SGA is effective and had a higher positive rate in screening malnutrition for patients with cancer. The NRS 2002 is not necessary for patients who are to be assessed with the PG-SGA.

## 1 Introduction

Cancer can lead to metabolic and physiological changes that result in severe malnutrition (1). The prevalence of malnutrition in patients with cancer has been reported more than 20% in worldwide studies (2–4). Accurate assessment and scientifically-guided nutritional intervention are critical for cancer patients (1). However, there is no consensus on the optimal approach to nutritional screening and assessment in this population (5). The Nutrition Risk Screening 2002 (NRS 2002) and the Patient-Generated Subjective Global Assessment (PG-SGA) are two commonly used nutritional screening tools in cancer settings, but they differ substantially in components and derivation (6, 7). The NRS 2002 was recommended by the Global Leadership Initiative on Malnutrition (GLIM) for initial screening (8). It considers disease severity, nutritional status, and age (9). In contrast, the PG-SGA was designed specifically for cancer populations. The PG-SGA consists of a patient self-assessment on weight, intake, symptoms, function, and a professional evaluation of metabolic needs, and a physical exam (10). It not only screens risk but also assesses current nutritional status and predicts clinical outcomes (11–13). Given the distinct nature of the tools, there is uncertainty regarding the necessity and added value of using the NRS 2002 together with the PG-SGA for nutritional screening in cancer patients (5). Some guidelines advocate adopting the PG-SGA as the singular approach (14, 15), while others recommend utilizing both (8). This study aimed to evaluate whether the PG-SGA alone can replace combined use with the NRS 2002 in cancer patients. Clarifying the optimal strategies for nutritional screening will enable targeted, effective interventions to improve patient nutrition and outcomes.

## 2 Materials and methods

### 2.1 Patients

This retrospective study collected data on cancer patients admitted from 2011 to 2018 at the First Affiliated Hospital of Jilin University. The NRS 2002 and the PG-SGA were performed within 48 h of admission. The NRS 2002 combines disease severity, nutritional score, and age adjustment, with a total score ≥3 indicating risk (4). The PG-SGA has patient-reported intake, symptoms, function, weight loss scores and professional-rated disease, stress, and exam scores. Total score ≥2 defined risk (16). Clinical characteristics, labs, handgrip strength (HGS), and appendicular skeletal muscle mass index (ASMI) were collected. Nutritional risk by the NRS 2002 and the PG-SGA were compared. The prognosis of non-risk groups was analyzed. No specific selection criteria were established for cancer type or demographic characteristics, except for patients who declined to participate in the study. The inclusion criteria were as follow:

1. Patients>65 y of age;
2. Pathologically diagnosed with malignant tumor; and
3. The Nutrition Risk Screening 2002 and the Patient-Generated Subjective Global Assessment completed within 48 h after admission.
4. No nutritional support treatment prior to nutritional assessment and laboratory testing.

Exclusion criteria included the following:

1. Patients who had two or more coexisting types of tumors;
2. Those who had incomplete records of necessary indexes.
3. Patients who died within 3 d after admission.

### 2.2 Measurements

Clinical-pathological variables include age, sex, weight, height, BMI, tumor types, TNM stages (AJCC 7th edition), and Karnofsky Performance Status (KPS). Laboratory examination results including total protein (TP), albumin, prealbumin (PAB), transferrin (TFN), C-reaction protein (CRP), neutrophil to lymphocyte ratio (NLR) and platelets to lymphocyte ratio (PLR) were collected. Anthropometric indices including hand-grip strength (HGS) and appendicular skeletal muscle mass index (ASMI) were measured by bioelectrical impedance analysis.

HGS was examined in all subjects using a Jamar hydraulic grip dynamometer (Sammons Preston Rolyan, Illinois, United States). Patients were comfortably seated in an upright position with the shoulders tucked in, neutral rotation, 90° elbow flexion, and the forearms and wrists in a neutral position. The patient gripped the dynamometer with maximum strength. The test is performed three times in a row, with a 1-min rest at the end of each set, and the maximum grip strength is recorded.

The skeletal muscle mass (SMM) was examined by the multifrequency bioelectrical impedance body composition analyzer InbodyS10 (Biospace Co., Seoul, Korea). Patients were required to empty their bladder, fast for 2 h, and rest before the measurement. Patients were asked to wear light clothes and were contacted with eight electrodes during the measurements. ASM was the sum of SMM in four limbs according to the formula of InbodyS10. The appendicular skeletal muscle mass index was calculated by ASM/height(m).

### 2.3 Nutritional risk assessment

The NRS 2002 and the PG-SGA nutritional assessments were completed within 48 h of patient admission.

**Figure 1:**
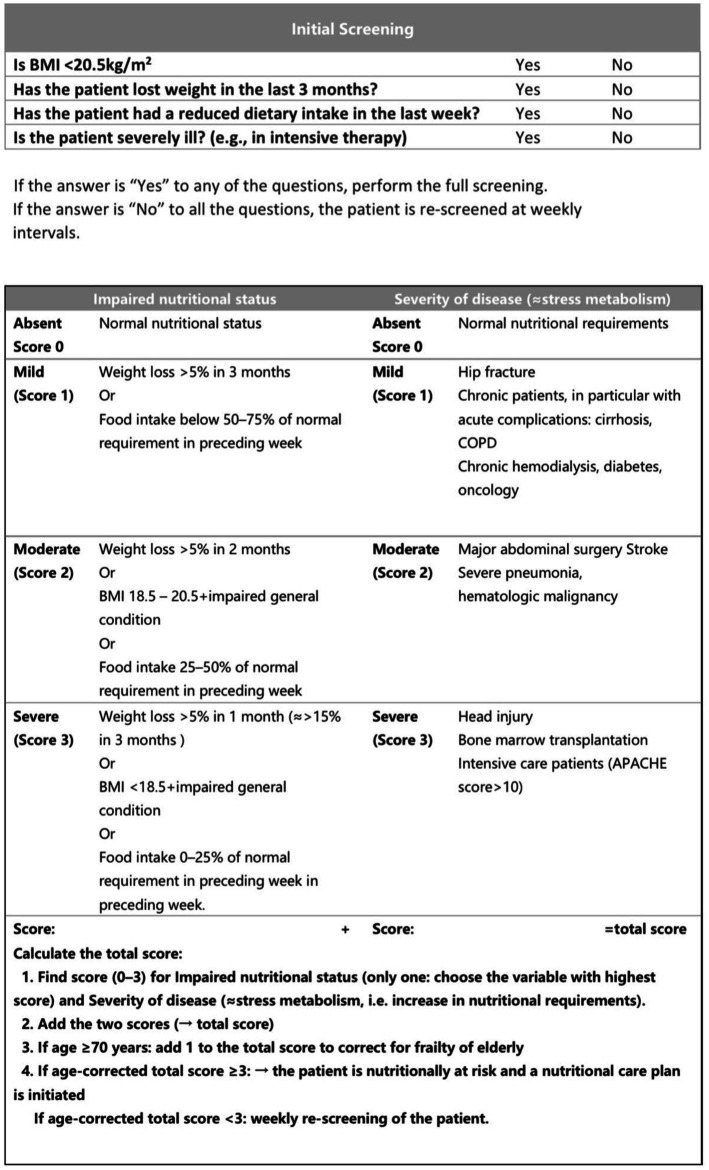
The NRS 2002 rating form.

In particular, the NRS 2002 total score consisted of the sum of three components, i.e., Disease Severity Score + Nutritional Status Hypoplasia Score + Age Score. A total score of ≥3 indicates that the patient is at nutritional risk (5). The scoring criteria are shown in Figure 1.

**Figure 2:**
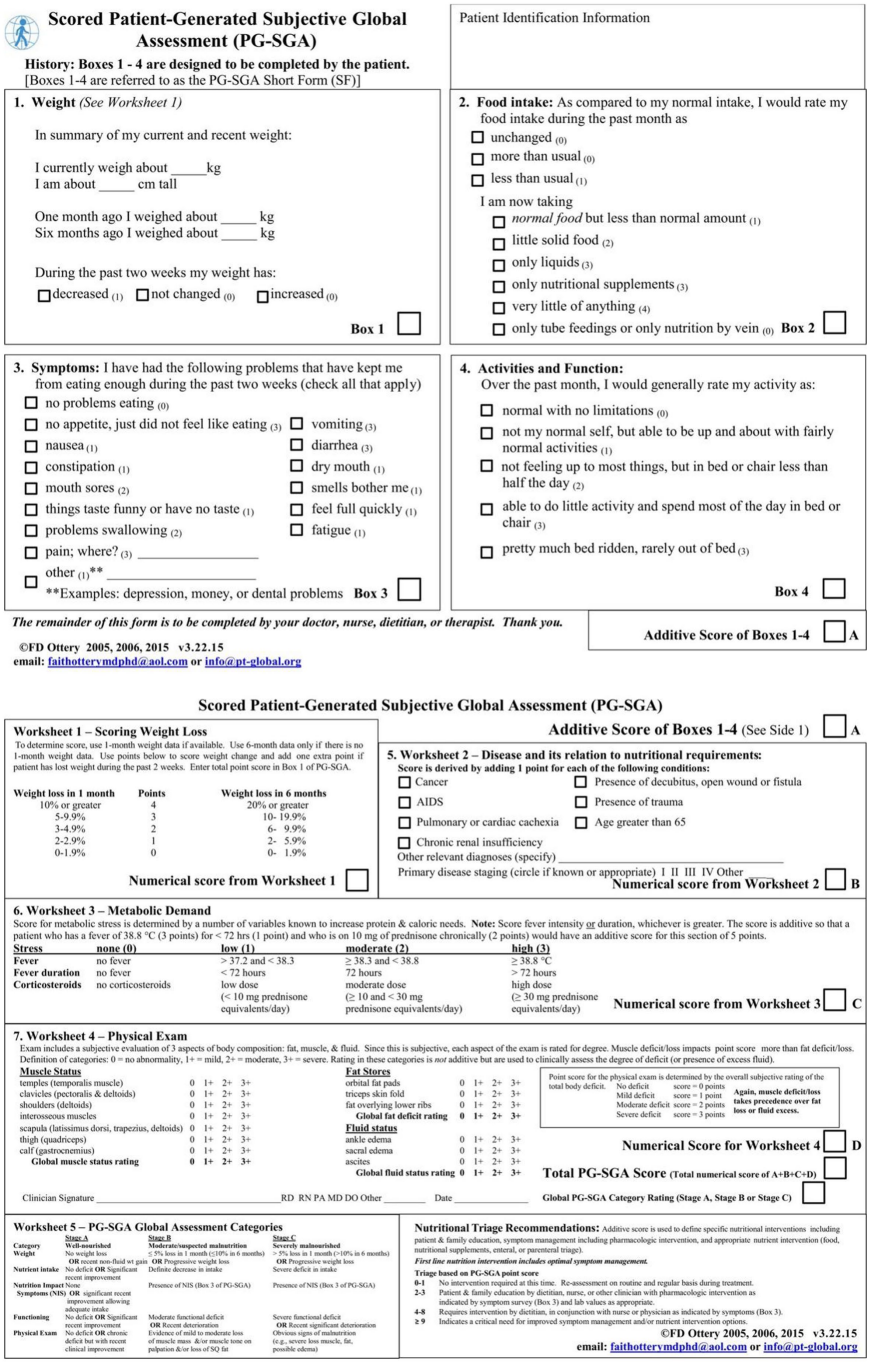
The PG-SGA rating form.

The PG-SGA begins with a self-assessment form (A score) completed by the patient, which includes four dimensions: body mass, feeding status, symptoms, activity, and physical function. Among them, the highest score option was selected for this score for feeding situation, and the symptoms were cumulative scores. The relationship between disease and nutritional requirements, metabolic needs and physical examination were then assessed by the medical staff. Patients’ disease status (B score) and stress status (C score) were cumulative scores, and physical examination (D score) determined patients’ fat, muscle, and fluid sub-item scores according to most parts of the body, and the muscle loss score was used as the final score for the physical examination items. The total A-D 4-item scores were summed, and a score of ≥2 was defined as being at nutritional risk. The scoring criteria are shown in Figure 2.

### 2.4 Statistical analysis

Analysis was performed using SPSS 26.0 statistical software. Data normality and chi-squareness were verified by the one-sample Kolmogorov - Smirnov test. Continuous variables that were normally distributed were expressed as mean ± standard deviation (x bar ± s), and comparisons between groups were made using independent samples *t*-tests. Continuous variables that did not follow a normal distribution after data transformation were expressed as median (interquartile range) and compared between groups using the two-sample Kolmogorov–Smirnov test. Categorical variables were expressed as percentages, and two or more groups of unordered categorical variables were tested using the χ^2^ test. *p* < 0.05 was regarded as statistically significant.

## 3 Results

### 3.1 Incidence of nutritional risk

**Figure 3:**
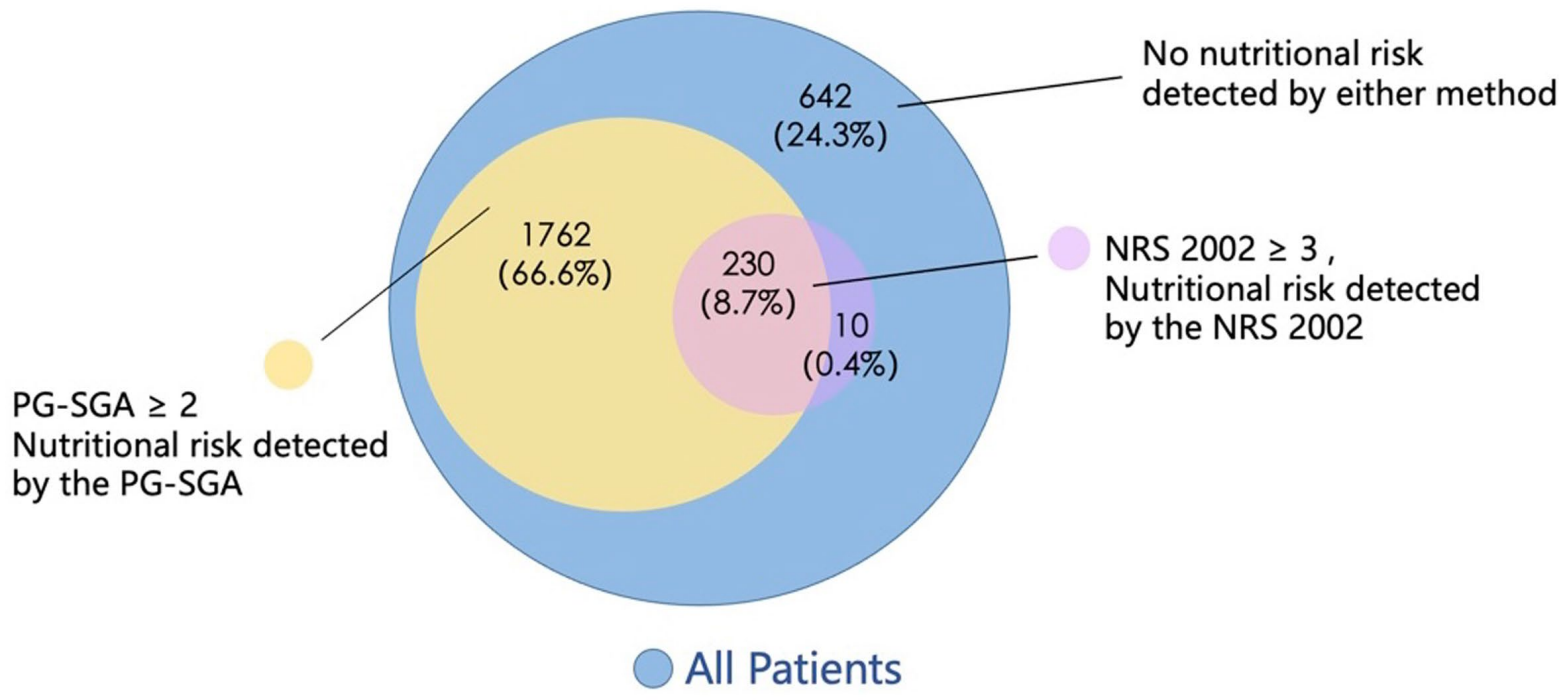
Patients’ nutritional risk detected by the NRS 2002 and the PG-SGA.

A total of 2,645 patients with cancer were included in the analysis. Two hundred and forty cases were screened with nutritional risk by the NRS 2002 (NRS ≥ 3 points) and 1993 cases were screened with nutritional risk by the PG-SGA (PG-SGA ≥ 2 points), and the incidence rates of malnutrition risk were 9.1 and 66.6%, respectively, as shown in Figure 3. The incidence rates of nutritional risk for different genders, ages, tumor types, and tumor stages screened by the two screening methods had a statistically significant (*p* < 0.05). The malnutrition incidence was higher in older patients and patients with advanced tumors, and patients with digestive tumors were more likely to be screened for nutritional risk (*p* < 0.05), as shown in Table 1.

**Table 1:** Comparison of the incidence of nutritional risk in different general conditions tumor patients.

Item	Number of patients	Nutritional risk screened by NRS 2002	Nutritional risk screened by PG-SGA	Chi-square value of NRS 2002	*p* value of NRS 2002	Chi-square value of PG-SGA	*p* value of PG-SGA
**Gender**				6.170	0.013	15.095	<0.001
Male	1,155	123 (10.7%)	913 (79.1%)				
Female	1,490	117 (7.9%)	1,080 (72.5%)				
**Age**							
<65	2,120	172 (8.1%)	1,469 (69.3%)	11.944	0.001	209.345	<0.001
>65	525	68 (12.9%)	524 (99.8%)				
Tumor type				59.100	<0.001	110.227	<0.001
Lung cancer	828	74 (8.9%)	618 (74.6%)				
Cancer of the digestive system	687	101 (14.7%)	605 (88.1%)				
Hematological malignancy	290	22 (7.6%)	200 (69.0%)				
Breast cancer	624	19 (3.0%)	400 (64.1%)				
Gynecological cancer	157	14 (8.9%)	123 (78.3%)				
Others	59	10 (16.9%)	47 (79.7%)				
Tumor staging				15.752	0.001	9.396	0.024
I	455	23 (5.1%)	326 (71.6%)				
II	580	51 (8.8%)	433 (74.7)				
III	659	79 (12.0%)	523 (79.4%)				
IV	951	87 (9.1%)	711 (74.8%)				

### 3.2 Comparison of the NRS-2002 and the PG-SGA screening results

Nutrition-related indicators of patients screened for malnutrition risk by both the NRS-2002 and the PG-SGA and those screened for malnutrition risk by the NRS-2002 were analyzed, and there were no statistically significant differences between the two populations in terms of grip strength, albumin, and muscle index, as shown in Table 2.

**Table 2:** Basic clinical information for all the NRS 2002 positive patients stratified by the PG-SGA positive.

Factors	PG-SGA, NRS 2002 positive (*N* = 230)	NRS 2002 positive (*N* = 240)	*p* value
KPS	87.63	88.00	0.748
TP (g/L)	64.58	64.65	0.901
Albumin (g/L)	36.82	36.95	0.993
PAB (g/L)	0.185	0.188	0.838
TFN (g/L)	2.22	2.24	0.811
HGS (kg)	22.44	22.73	0.791
ASMI (kg/m^2^)	5.98	5.98	0.837

KPS, karnofsky performance status; TP, total protein; PAB, prealbumin; TFN, transferrin; HGS, hand-grip strength; ASMI, appendicular skeletal muscle mass index.

### 3.3 Prognosis of patients screened for malnutrition by the NRS-2002 vs. the PG-SGA

**Figure 4:**
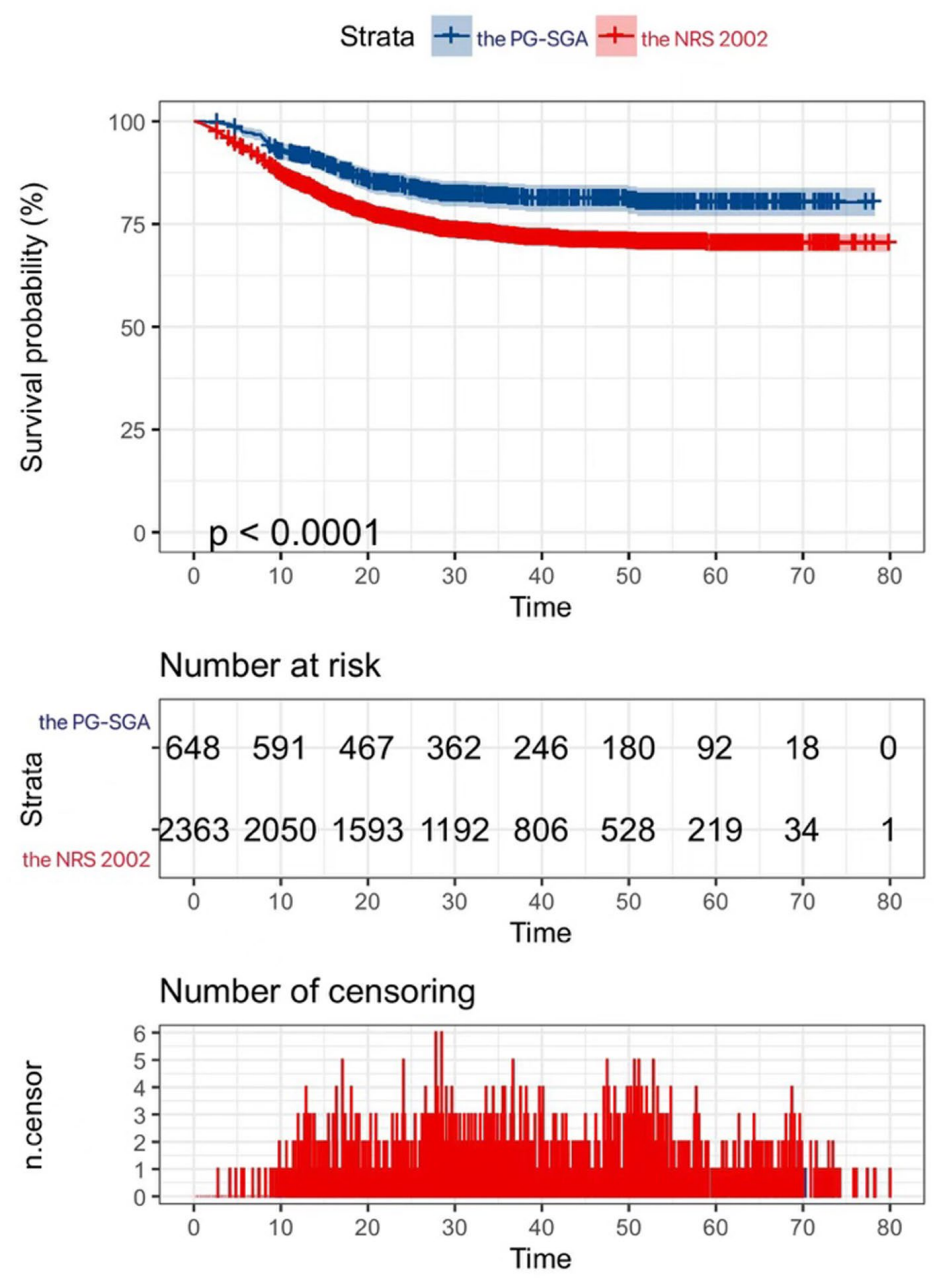
|Kaplan–Meier curves for patients without nutritional risk.

Prognostic analyses of those not screened for nutritional risk by the NRS-2002 and those not screened for nutritional risk by the PG-SGA were performed, and the results of the survival analyses showed that the prognosis of those not screened for nutritional risk by the PG-SGA was better than that of those not screened for nutritional risk by the NRS 2002 (*p* < 0.05), as shown in Figure 4.

## 4 Discussion

Many patients with cancer are at risk for malnutrition and nutritional assessment is a vital aspect of cancer care (17). While nutrition risk screening tools contribute to the early recognition of malnutrition, nutritional screening and assessment are established in many oncological clinical settings. The NRS 2002 is recommended as the screening tool before malnutrition diagnosis according to the global leadership initiative on malnutrition (5), while the PG-SGA is recommended in various national guidelines for nutrition in patients with cancer (15). Studies showed that the PG-SGA is not only an assessment that identifies existing malnutrition, but it can also be used as a screening instrument for nutritional risk or deficit (10).

The PG-SGA identified more patients at nutritional risk than the NRS 2002 in this study. Among patients not classified as high risk by the NRS 2002, those additionally identified as malnourished by the PG-SGA had poorer survival. The PG-SGA non-risk group had a better prognosis than the NRS 2002 non-risk, suggesting the NRS 2002 may miss patients with risk. Our study indicates the PG-SGA screens cancer patients more comprehensively for nutritional risk than the NRS 2002.

### 4.1 Key findings and significance

The much higher prevalence of nutritional risk by the PG-SGA implies it is more sensitive than the NRS 2002 in this population (12, 18). This aligns with prior studies demonstrating higher detection rates of malnutrition by the PG-SGA vs. other single-item screening methods (19). The PG-SGA’s detailed capture of reduced intake and aggravated cancer symptoms underpins its greater sensitivity over the NRS 2002’s reliance on primarily weight loss and disease severity (20, 21). Though the NRS 2002 is recommended for initial screening, it appears insufficient on its own based on the poorer outcomes of patients not identified as at-risk. This finding indicates sole use of the NRS 2002 risks overlooking some patients who need and could benefit from early nutritional intervention (6, 22). Using the PG-SGA additionally would allow more complete detection of patients requiring intervention. This highlights the necessity of multi-modal nutritional screening in cancer patients to avoid overlooking opportunities for important nutritional support (7, 15).

This study confirms the high sensitivity of the PG-SGA as a nutritional risk screening tool optimized specifically for cancer patients. Its cancer-specific derivations such as patient-reported symptom impact likely underlie its superior prognostic utility over the generic NRS 2002, supporting its preferential use for prospectively assessing malnutrition risk in this population (10, 14). Thorough nutritional screening is crucial for providing appropriate supportive care to improve patient nutrition and outcomes.

### 4.2 Possible reasons for differences in screening tools

The NRS 2002 was designed for hospital inpatients generally, while the PG-SGA was optimized specifically for cancer populations. The PG-SGA’s cancer-specific design underlies its superior sensitivity in detecting nutritional risk over the more generic NRS 2002. The PG-SGA captures extensive information about reduced oral intake and symptom impacts like anorexia, nausea, vomiting, and dysphagia that are highly prevalent in cancer patients but not addressed in the NRS 2002 (14, 15). The inclusion of patient self-assessment also enhances its sensitivity, which provides unique subjective data on changes in weight, food intake, and functional capacity that clinicians cannot observe as accurately (10). These key components enhance early identification of malnutrition before severe manifestations appear. Furthermore, the PG-SGA categorizes risk level of malnutrition severity rather than just presence/absence (23). This differentiation allows nutrition interventions to be personalized and scaled based on the grade of malnutrition. The multidimensional nature of the PG-SGA makes it better suited to the complex etiology of cancer cachexia compared to the NRS 2002.

### 4.3 Limitations and future research

As a single-center retrospective study, the results may not generalize to other cancer populations. Additional studies should validate findings in other geographical and ethnic groups (24). Future research could also compare the PG-SGA to other cancer-specific tools like the Malnutrition Screening Tool (MST) to determine optimal approaches for different oncology settings (25). Incorporating nutritional biomarkers may provide further objective insight into differences between patients classified as non-risk by each tool (26). Most importantly, future research should investigate the impacts of PG-SGA screening on clinically meaningful outcomes like treatment response, quality of life, and survival with nutritional interventions (27). Cost-effectiveness analyses will also inform implementation. Ultimately, determining optimal nutritional assessment strategies will require investigations into the impacts on patient-important outcomes with nutritional interventions through high-quality randomized controlled trials (28).

## 5 Conclusion

In cancer patients, the PG-SGA provides more comprehensive nutritional risk detection than the NRS 2002, with prognostic utility. Relying solely on the NRS 2002 risks overlooking at-risk patients who may benefit from nutrition support. The high sensitivity of the PG-SGA underscores its value for identifying malnourishment requiring intervention in cancer populations. Clinicians should be aware that patients classified as non-risk by the NRS 2002 may still be at nutrition-related risk detectable by the in-depth PG-SGA. Implementing the PG-SGA’s cancer-specific approach is vital for optimal nutritional risk screening and assessment in oncology settings. The NRS 2002 is not necessary for patients who are to be assessed with the PG-SGA.

## Data availability statement

The original contributions presented in the study are included in the article/supplementary material, further inquiries can be directed to the corresponding author.

## Ethics statement

The studies involving humans were approved by First affiliated hospital of Jilin University. The studies were conducted in accordance with the local legislation and institutional requirements. The participants provided their written informed consent to participate in this study.

## Author contributions

XC: Formal analysis, Investigation, Methodology, Writing – original draft. XL: Conceptualization, Funding acquisition, Methodology, Resources, Supervision, Validation, Writing – review & editing. WJ: Conceptualization, Data curation, Investigation, Writing – review & editing. YZ: Data curation, Formal analysis, Methodology, Writing – review & editing. YH: Data curation, Formal analysis, Methodology, Writing – review & editing. YL: Data curation, Investigation, Software, Writing – review & editing. QL: Data curation, Formal analysis, Software, Writing – review & editing. HS: Project administration, Supervision, Writing – review & editing. JC: Project administration, Resources, Supervision, Writing – review & editing.

## References

[1] Arends J Baracos V Bertz H Bozzetti F Calder PC Deutz NEP. ESPEN expert group recommendations for action against cancer-related malnutrition. Clin Nutr. (2017) 36:1187–96. doi: 10.1016/j.clnu.2017.06.017, PMID: 28689670

[2] Planas M Álvarez-Hernández J León-Sanz M Celaya-Pérez S Araujo K García de Lorenzo A. Prevalence of hospital malnutrition in cancer patients: a sub-analysis of the PREDyCES® study. Support Care Cancer. (2016) 24:429–35. doi: 10.1007/s00520-015-2813-7, PMID: 26099900

[3] Hébuterne X Lemarié E Michallet M de Montreuil CB Schneider SM Goldwasser F. Goldwasser prevalence of malnutrition and current use of nutrition support in patients with cancer. J Parenter Enter Nutr. (2014) 38:196–204. doi: 10.1177/0148607113502674 24748626

[4] Silva FR de Oliveira MG Souza AS Figueroa JN Santos CS. Santos factors associated with malnutrition in hospitalized cancer patients: a cross-sectional study. Nutr J. (2015) 14:123. doi: 10.1186/s12937-015-0113-1, PMID: 26652158 PMC4676158

[5] Cederholm T Jensen GL Correia MITD Gonzalez MC Fukushima R Higashiguchi T. GLIM criteria for the diagnosis of malnutrition – a consensus report from the global clinical nutrition community. Clin Nutr. (2019) 38:1–9. doi: 10.1016/j.clnu.2018.08.002, PMID: 30181091

[6] Kondrup J Rasmussen HH Hamberg O Stanga Z Ad Hoc ESPEN Working Group. Nutritional risk screening (NRS 2002): a new method based on an analysis of controlled clinical trials. Clin Nutr. (2003) 22:321–36. doi: 10.1016/S0261-5614(02)00214-5, PMID: 12765673

[7] Ottery FD. Definition of standardized nutritional assessment and interventional pathways in oncology. Nutrition. (1996) 12:S15–9. doi: 10.1016/0899-9007(95)00067-4, PMID: 8850213

[8] Cederholm T Barazzoni R Austin P Ballmer P Biolo G Bischoff SC. ESPEN guidelines on definitions and terminology of clinical nutrition. Clin Nutr. (2017) 36:49–64. doi: 10.1016/j.clnu.2016.09.004, PMID: 27642056

[9] Kondrup J Allison SP Elia M Vellas B Plauth M. Educational and clinical practice committee, European Society of Parenteral and Enteral Nutrition (ESPEN). ESPEN guidelines for nutrition screening 2002. Clin Nutr. (2003) 22:415–21. doi: 10.1016/S0261-5614(03)00098-0, PMID: 12880610

[10] Jager-Wittenaar H Ottery FD. Assessing nutritional status in cancer: role of the patient-generated subjective global assessment. Curr Opin Clin Nutr Metab Care. (2017) 20:322–9. doi: 10.1097/MCO.0000000000000389 28562490

[11] Isenring E Bauer J Capra S. The scored patient-generated subjective global assessment (PG-SGA) and its association with quality of life in ambulatory patients receiving radiotherapy. Eur J Clin Nutr. (2003) 57:305–9. doi: 10.1038/sj.ejcn.1601552, PMID: 12571664

[12] Hogan SE Solomon MJ Carey SK. Exploring reasons behind patient compliance with nutrition supplements before pelvic exenteration surgery. Support Care Cancer. (2019) 27:1853–60. doi: 10.1007/s00520-018-4445-1, PMID: 30187221

[13] Cotogni P De Carli L Passera R Amerio ML Agnello E Fadda M. Longitudinal study of quality of life in advanced cancer patients on home parenteral nutrition. Cancer Med. (2017) 6:1799–806. doi: 10.1002/cam4.1111, PMID: 28557362 PMC5504329

[14] Arends J Bachmann P Baracos V Barthelemy N Bertz H Bozzetti F. ESPEN guidelines on nutrition in cancer patients. Clin Nutr. (2017) 36:11–48. doi: 10.1016/j.clnu.2016.07.015 27637832

[15] Talwar B Donnelly R Skelly R Donaldson M. Nutritional management in head and neck cancer: United Kingdom National Multidisciplinary Guidelines. J Laryngol Otol. (2016) 130:S32–40. doi: 10.1017/S0022215116000402, PMID: 27841109 PMC4873913

[16] Henriksen C Paur I Pedersen A Kværner AS Ræder H Henriksen HB. Agreement between GLIM and PG-SGA for diagnosis of malnutrition depends on the screening tool used in GLIM. Clin Nutr. (2022) 41:329–36. doi: 10.1016/j.clnu.2021.12.024, PMID: 34999327

[17] Jiuwei C Yudi L Fangqi L Wenlei Z Lan H Chuan X. Evidence-based guideline on immunonutrition in patients with cancer. Precision Nutr. (2023) 2:e00031. doi: 10.1097/PN9.0000000000000031

[18] Thoresen L Frykholm G Lydersen S Ulveland H Baracos V Prado CMM. Nutritional status, cachexia and survival in patients with advanced colorectal carcinoma. Different assessment criteria for nutritional status provide unequal results. Clin Nutr. (2013) 32:65–72. doi: 10.1016/j.clnu.2012.05.009, PMID: 22695408

[19] Cereda E Cappello S Colombo S Klersy C Imarisio C Turri A. Nutritional counseling with or without systematic use of oral nutritional supplements in head and neck cancer patients undergoing radiotherapy. Radiother Oncol. (2018) 126:81–8. doi: 10.1016/j.radonc.2017.10.015 29111172

[20] Silander E Nyman J Hammerlid E. An exploration of factors predicting malnutrition in patients with advanced head and neck cancer. Laryngoscope. (2013) 123:2428–34. doi: 10.1002/lary.23877, PMID: 23918730

[21] Blackburn GL Bistrian BR Maini BS Schlamm HT Smith MF. Nutritional and metabolic assessment of the hospitalized patient. JPEN J Parenter Enteral Nutr. (1977) 1:11–21. doi: 10.1177/014860717700100101 98649

[22] Detsky AS McLaughlin JR Baker JP Johnston N Whittaker S Mendelson RA. What is subjective global assessment of nutritional status? JPEN J Parenter Enteral Nutr. (1987) 11:8–13. doi: 10.1177/014860718701100108 3820522

[23] Leuenberger M Kurmann S Stanga Z. Nutritional screening tools in daily clinical practice: the focus on cancer. Support Care Cancer. (2010) 18:17–27. doi: 10.1007/s00520-009-0805-1 20087607

[24] Norman K Pichard C Lochs H Pirlich M. Prognostic impact of disease-related malnutrition. Clin Nutr. (2008) 27:5–15. doi: 10.1016/j.clnu.2007.10.007, PMID: 18061312

[25] Ferguson M Capra S Bauer J Banks M. Development of a valid and reliable malnutrition screening tool for adult acute hospital patients. Nutrition. (1999) 15:458–64. doi: 10.1016/S0899-9007(99)00084-2, PMID: 10378201

[26] Bozzetti F. Screening the nutritional status in oncology: a preliminary report on 1,000 outpatients. Support Care Cancer. (2009) 17:279–84. doi: 10.1007/s00520-008-0476-3, PMID: 18581148

[27] Ferry M. Strategies for ensuring good hydration in the elderly. Nutr Rev. (2005) 63:S22–9. doi: 10.1111/j.1753-4887.2005.tb00151.x, PMID: 16028569

[28] Baldwin C Spiro A Ahern R Emery PW. Oral nutritional interventions in malnourished patients with cancer: a systematic review and meta-analysis. J Natl Cancer Inst. (2012) 104:371–85. doi: 10.1093/jnci/djr556, PMID: 22345712

